# Transosseous suture versus suture anchor fixation for inferior pole fractures of the patella in osteoporotic bone: a biomechanical study

**DOI:** 10.1186/s40001-022-00903-9

**Published:** 2022-12-03

**Authors:** Jana Seggewiss, Luis Fernando Nicolini, Philipp Lichte, Johannes Greven, Marx Ribeiro, Andreas Prescher, Roman Michalik, Christian Herren, Philipp Kobbe, Frank Hildebrand, Miguel Pishnamaz

**Affiliations:** 1 Department of Orthopaedics, Trauma and Reconstructive Surgery, RWTH Aachen University Hospital, Pauwelsstr. 30, 52074 Aachen, Germany; 2 Institute of Molecular and Cellular Anatomy, RWTH Aachen University Hospital, Pauwelsstr. 30, 52074 Aachen, Germany; 3 Fontanestr. 57, 47877 Willich, Germany; 4 Institute of General Mechanics (IAM), RWTH Aachen University, Eilfschornsteinstr. 18, 52062 Aachen, Germany

**Keywords:** Patella fracture, Suture anchor, Transosseous suture, Biomechanical, Osteoporotic fracture, Bone mineral density

## Abstract

**Background:**

The surgical treatment of inferior patellar pole fractures can be a challenge, especially in geriatric patients, who are particularly frequently affected by osteoporosis. The objective of this biomechanical study was to compare the performance of suture anchor and transosseous suture fixation in fractures of the inferior patellar pole in context of bone mineral density.

**Methods:**

Twelve fresh-frozen human cadaveric knees received a transverse osteotomy, simulating an AO/OTA 34C1.3 inferior pole fracture of the patella. These fractures were fixated with either suture anchors (SA; Corkscrew^®^ FT 4.5 mm) or transosseous suture (TS; #2 FiberWire^®^). Cyclic loading tests were performed by pulling the quadriceps tendon against gravity from 90° flexion to almost full extension (5°) for 1000 cycles. Motion and fracture gap displacement were tracked until failure occurred. Subsequently, loading to failure tests followed. Differences between groups were compared using unpaired *t*-tests, and correlations were calculated with Pearson’s correlation coefficient.

**Results:**

The suture anchor group showed significantly fewer cycles to failure than the transosseous suture group (SA: 539.0 ± 465.6 cycles, TS: 1000 ± 0 cycles, *P* = 0.04). Bone mineral density correlated positively with cycles to failure in the suture anchor group (Pearson’s *r* = 0.60, *P* = 0.02). No differences in fracture gap displacement could be proven after 100 cycles (SA: 4.1 ± 2.6 mm, TS: 6.5 ± 2.6 mm, *P* = 0.19); 500 cycles (SA: 6.4 ± 6.1 mm, TS: 9.6 ± 3.8 mm, *P* = 0.39); and 1000 cycles (SA: 4.0 ± 0.4 mm, TS: 11.0 ± 4.5 mm, *P* = 0.08). Furthermore, the mean destructive load to failure in the suture anchor group was also significantly lower than in the transosseous suture group (SA: 422.4 ± 212.2 N, TS: 825.7 ± 189.3 N, *P* = 0.04).

**Conclusions:**

Suture anchors may be a viable alternative to transosseous suture in younger patients for clinical advantages, but in osteoporotic bone, the more stable osteosynthesis with transosseous suture continues to prove superior. Therefore, trauma surgeons might consider the use of transosseous suture in elderly patients, especially in those presenting with low bone mineral density values.

## Background

As the largest sesamoid bone of the human body, the patella protects the quadriceps tendon where it slides across the distal femur [1] and thereby increases the knee extensors’ moment arm up to 30% by extending the virtual lever arm of the quadriceps muscle at the knee joint [2, 3]. Consequently, surgery is required in all displaced fractures with impaired extensor function [3, 4]. Fractures of the patella encompass approximately 1% of all human body fractures and 9.3–22.4% of those are categorized as inferior pole fractures [5, 6]. Common injury mechanisms involve direct forces like a fall or dashboard injury or indirect forces like excessive tension by the quadriceps muscle [3, 6].

For fractures of the distal patella pole (AO/OTA 34C1.3) [7], common surgical techniques are transosseous suture (TS) repair, tension band stitch, or wiring [4, 8]. Even though tension band techniques have been the gold standard procedures for decades, especially in simple transverse patella fractures, their high re-operation rates owing to loosening of the material have scope for improvement. The TS avoids this problem and can also be adapted more individually to complicated and possibly comminuted inferior pole fractures [8, 9]. Suture anchor fixation (SA) is an alternative procedure, presenting advantages such as a less invasive approach, particularly in the region of the superior edge of the patella and the insertion area of the quadriceps tendon; decreased surgical time; and no need for material removal [10, 11]. Previously established for surgeries like patella and quadriceps tendon reattachment, the SA fixation has already shown some promising clinical results [5, 11–13].

Although many human cadaveric biomechanical studies on the instrumentation of transverse patella fractures have been published [14–23], only three publications thus far have investigated fractures of the inferior patella pole [24–26]. With increased interest in the use of suture anchors in the treatment of inferior pole patella fractures recently, the latest publication by O’Donell et al. [24] also investigated the use of SA or TS in extraarticular inferior pole patella fractures but with partial patellectomy in a biomechanical setup with non-osteoporotic human patellae and patellar tendons.

Given that we are experiencing an age shift in the overall population, the number of geriatric patients will increase in the future. The incidence of fractures in osteoporotic bone will therefore increase, based on the association between patient age and osteoporosis [27]. Therefore, surgical techniques that can cope with osteoporotic bone are becoming more important.

We aimed to evaluate the biomechanical performance of SA and TS in fractures of the inferior patellar pole, especially in relation to bone mineral density (BMD). We hypothesized that SA would endure a similar number of cycles to failure and fracture gap displacement during cyclic loading tests and similar destructive loads to failure, compared to the standard TS technique.

## Methods

### Specimens and preparation

Twelve fresh-frozen human cadaveric knees from 5 male and 3 female donors with a mean age of 78.8 ± 13.4 years at the time of death were included in this study. There were 4 pairs of knees and 4 single knees. Physiological range of motion was assessed by physical examination. The BMD of each patella was estimated by using a procedure described by Schreiber et al. [28]. The knee pairs were split between the two testing groups and the other specimens were allocated according to age, sex, and BMD. This was done to minimize differences between the groups. Each group then consisted of four male and two female specimens as well as three left and three right knees.

Each specimen was dissected of soft tissue. Subsequently, the knee joint capsule, ligaments, and extensor mechanism were carefully visualized [16]. The femora were shortened 16 cm proximally and the tibiae 13 cm distally to the knee joint. The transverse osteotomy was performed at a 1 cm distance from the inferior patella pole with a handsaw (1 mm thickness) to simulate an AO/OTA 34C1.3 fracture [7]. The proximal 6 cm of the femora were embedded in polymethylmethacrylate (PMMA) (Technovit^®^ 3040, Kulzer GmbH, Hanau, Germany) [16]. The knee joints were stored at − 18 °C and thawed overnight at 8 °C prior to surgery and testing [21].

### Instrumentation

SA group: the suture anchor procedure was performed by an experienced surgeon according to the Kadar et al.’s protocol [5]. At first, two holes were drilled in the middle of each fragment 1 cm apart from each other to implement the anchors (Corkscrew^®^ FT, Suture Anchor 4.5 × 14 mm, Titanium, with #2 FiberWire, Arthrex GmbH, Munich, Germany) and guide their sutures through the lower fragment. A Krackow whipstitch with four loops was performed to pull the fragments together as tight as possible for anatomical repositioning. Finally, the sutures were tied together.

**Fig. 1:**
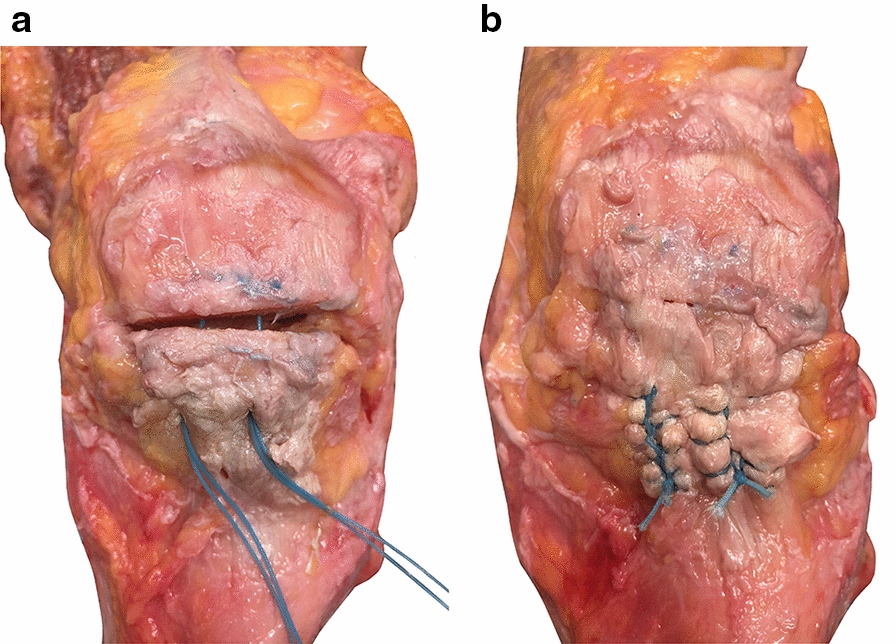
Transosseous suture technique in a human cadaveric specimen: sutures have been pulled through both fragments and stitched through the patella tendon (**a)**; Krackow whipstitches with four loops and surgeons knot were tied in the patella tendon (**b)**

TS group: for the transosseous suture technique, both fragments were drilled as described for the SA procedure; in this case however, the upper patella parts were drilled through completely. The two sutures (#2 FiberWire^®^, Arthrex GmbH, Munich, Germany) were first passed through one tunnel in the lower fragment, and then through the opposite tunnel in the upper fragment, stitching out of the quadriceps tendon and then back through the lower fragment, and again emerging at the patella tendon (Fig. 1a). To ensure good comparability between the two groups, similar Krackow whipstitches were performed in both the SA and TS groups (Fig. 1b).

### Biomechanical testing

**Fig. 2:**
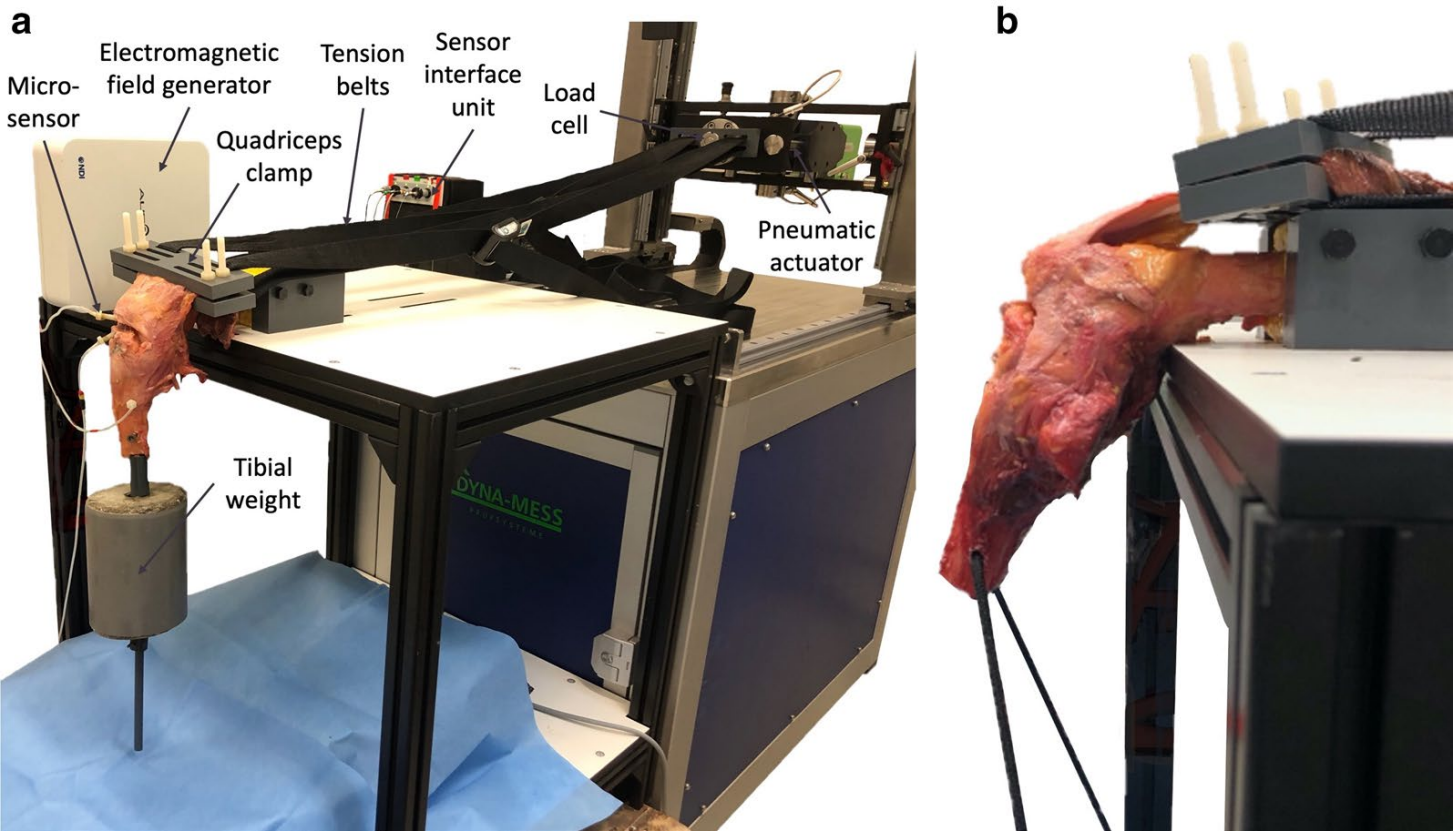
**a**: Setup for biomechanical cyclic loading tests of a human cadaveric knee. The femur is kept fixed while a load is applied on the quadriceps tendon via two tension belts and a quadriceps clamp to move the knee from 90° flexion (as shown here) to almost full extension against gravity. An electromagnetic tracking system captures the kinematics of microsensors inserted in the upper and lower patella fragment and the tibia. **b**: Setup for biomechanical load to failure tests with a human cadaveric knee. The specimen is fixated rigidly with its embedded femur and tied in a 45° position with a nylon rope through the tibia. The load is translated to the quadriceps tendon by the clamp and tension belts

Biomechanical tests were performed on a custom-built servo-pneumatic material testing machine (DYNA-MESS Prüfsysteme GmbH, Stolberg, Germany) with knee joint movement from 90° flexion to almost full extension against gravity by pulling the quadriceps tendon [14, 15, 17, 19, 21–23, 29, 30]. This simulates the natural course of movement in the recovery phase of a patient who underwent surgery for an inferior patellar pole fracture. The recommended limit of flexion in these cases is 90° until the 6th week of recovery same as for patella tendon ruptures [31]. The embedded part of the femur was fixed horizontally. In order to simulate the weight of the lower leg and foot, a 3-kg concrete cylinder was attached at 25 cm from the joint line to provide an equivalent moment load [14, 32]. The quadriceps tendon was grabbed by a size-adjustable clamp, so the tibia was allowed to move freely from 90° flexion to almost full extension (5°) by pulling the clamp with two inelastic tension belts attached to the actuator of the testing machine (Fig. 2a).

Each specimen was moved at a velocity of 5 mm/s up to the desired range of motion (5°), stopped, and let down manually to determine the individually required load associated with almost full extension. Subsequently, this load was applied as target load over 1000 flexion–extension cycles at a velocity of 20 mm/s or until failure occurred. The load applied to the quadriceps muscle was recorded by a load cell at a frequency of 100 Hz with a maximum error of 1% relative to the selected nominal load. An electromagnetic tracking system (Aurora^®^, NDI Europe GmbH, Radolfzell, Germany) was used to track the motion of microsensors glued to the patellar fragments and inserted into the tibia, whereby the exact cycle when failure occurred could be determined [33]. Additionally, the fracture gap was measured with a digital calliper (resolution: 0.01 mm, accuracy: ± 0.02 mm) every 20 cycles for the first 100 cycles and then every 50 cycles up to 1000 cycles [29, 34]. Only the values up to an optional failure were included in the Fracture Gap Data.

Subsequently, a load to failure protocol similar to the one described by Carpenter et al. [15] was performed. For this, the tibiae were fixed in a 45° position for maximum disruptive forces by threading a nylon rope through two holes in the tibial shaft (Fig. 2b) [15, 26, 30]. An increasing load was translated on the quadriceps tendon with a velocity of 5 mm/s until failure occurred.

For both cyclic loading and load to failure tests, the failure criterion was predefined as implant failure, fracturing of the patella, laceration of the patella or quadriceps tendon with no opportunity to refixate [15, 26, 30]. The morphology of implant failure was further categorized in implant pullout or rupture of suture. To avoid tissue dehydration, the specimens were kept moist during tests.

### Data analysis

All statistical analysis was performed using GraphPad Prism (Version 9.0.0, GraphPad Software, San Diego, USA) with the level of significance set to 0.05 for all tests. Unpaired *t*-tests were used to identify and compare differences in tensile loads for cyclic loading; number of cycles to failure; fracture gap displacement after 100, 500, and 1000 cycles; and destructive load to failure between the SA and TS groups. Pearson’s correlation coefficient was calculated for tensile load and age of the donors at death, BMD, and the number of cycles to failure in the SA group as well as BMD and destructive load to failure. An a priori power analysis was performed on previously published data by Ettinger et al. [35] for fracture gap displacement and load to failure, which showed that a sample size of 3 or 6 specimens in each group, respectively, should be adequate to obtain the desired power of 90% (G*Power, Heinrich-Heine-Universität, Düsseldorf, Germany [36]).

## Results

For the SA group, the mean age of the donors at death was 78.3 ± 13.7 years, and for the TS group, the mean age was 79.3 ± 14.4 years (*P* = 0.90). There was no significant difference in the BMD between the two testing groups (SA: 298.3 ± 89.6 HU, TS: 337.0 ± 96.1 HU, *P* = 0.49) as well as in the applied tensile loads during cyclic loading (SA: 336.7 ± 196.0 N, TS: 313.3 ± 94.4 N, *P* = 0.80).

### Cycles to failure

**Fig. 3:**
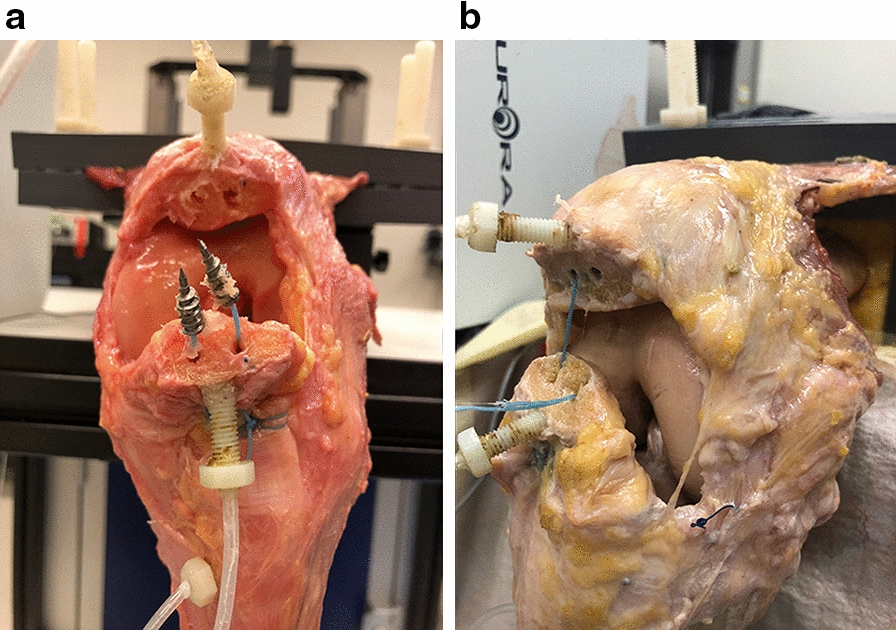
Failure mechanisms in suture anchors during cyclic loading: implant failure of human cadaveric knee specimen with distal pole patellar fractures instrumented with suture anchors (SA) during cyclic loading. **a**: Pullout of the suture anchors. **b**: Rupture of the sutures

The SA group showed significantly lesser resistance to cyclic loading than the TS group (number of cycles to failure SA: 539.0 ± 465.6 cycles, TS: 1000 ± 0 cycles, *P* = 0.04). Furthermore, in the SA group, 4/6 specimens did not withstand cyclic loading tests. Three of them failed by implant pullout (Fig. 3a), while in the remaining case, the suture ruptured (Fig. 3b). In contrast, all specimens in the TS group outlasted cyclic loading resulting in the full number of cycles.

**Fig. 4:**
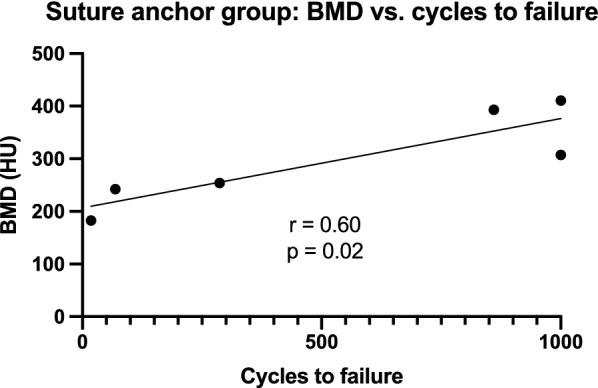
Suture anchor group—BMD vs. cycles to failure: distribution of the bone mineral density (BMD) versus the number of cycles performed until failure or a maximum of 1000 cycles with a simple linear regression curve and Pearson correlation coefficient (*r* = 0.60, *P* = 0.02). The specimens were treated with suture anchors after receiving a distal pole patellar fracture

The Pearson correlation coefficient of the BMD and cycles to failure in the SA group (Fig. 4) resulted in a statistically significant positive correlation (*r* = 0.60, *P* = 0.02).

### Fracture gap displacement

**Fig. 5:**
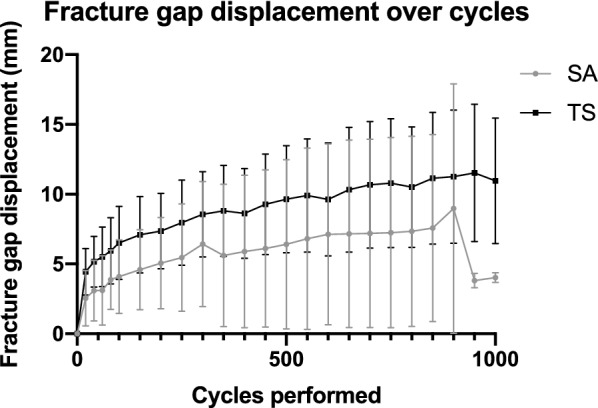
Fracture gap displacement over cycles: mean fracture gap displacement over cycles in the suture anchor (SA) and transosseous suture (TS) groups. The error bars represent the standard deviations. At 18, 69, 287 and 860 cycles one specimen each broke and therefore were no longer included in the calculation for the following points in cycles

No significant differences in fracture gap displacement were identified after 100 cycles (SA: 4.1 ± 2.6 mm, TS: 6.5 ± 2.6 mm, *P* = 0.19); 500 cycles (SA: 6.4 ± 6.1 mm, TS: 9.6 ± 3.8 mm, *P* = 0.39); and 1000 cycles (SA: 4.0 ± 0.4 mm, TS: 11.0 ± 4.5 mm, *P* = 0.08). However, the mean fracture gap displacement showed a trend of lesser displacement over all cycles in the SA group than in the TS group (Fig. 5).

### Load to failure

**Fig. 6:**
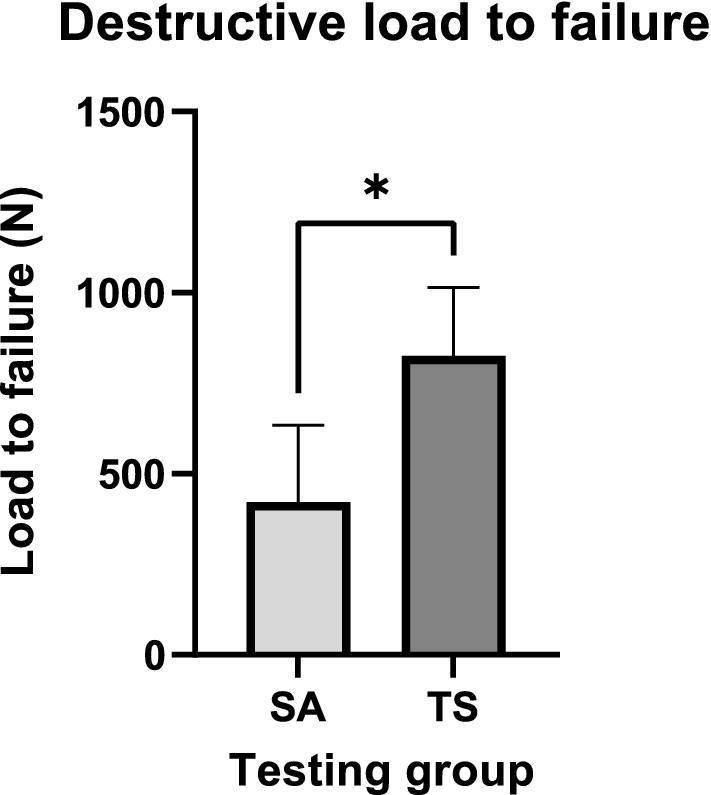
Destructive load to failure: mean load to failure measured in human cadaveric knees with distal pole patellar fracture instrumented with either suture anchors (SA) or transosseous suture (TS). The error bars represent the standard deviation

Mean destructive load to failure was significantly lower in the SA group than in the TS group (SA: 422.4 ± 212.2 N, TS: 825.7 ± 189.3 N, *P* = 0.04) as shown in Fig. 6. BMD did neither correlate with the destructive load to failure of all specimens (Pearson’s *r* = − 0.05, *P* = 0.45) nor of the TS group’s specimens (Pearson’s *r* = 0,2214, *P* = 0.336). A correlation between BMD and the destructive load to failure in the SA group could not be calculated due to low sample size.

In the SA group, the failure mode of the two remaining specimens was categorized as implant failure (implant pullout). In the TS group, one implant failure (rupture of suture) and five quadriceps tendon ruptures occurred.

## Discussion

The treatment of inferior patella pole fractures can be a surgical challenge, especially in geriatric patients. Various implants and techniques are available to stabilize the fracture elements, but a gold standard is not yet defined. Although SAs are more expensive than the TS, they require a smaller surgical access, no suture placement above the quadriceps tendon, and are easier to use with a better clinical outcome [5, 10–12]. SAs provide decreased surgical and tourniquet time [5, 10], in turn resulting in reduced costs, surgical morbidity, and complications [37]. To achieve optimal treatment results, consideration of biomechanical aspects is essential.

For the flexion–extension movements of a patient during the typical recovery time of 2–3 months after an operated patella fracture, the number of 100,000 cycles is considered as equivalent [34, 38]. Consistent with previous studies [29, 34], we measured the most substantial changes in both cycles to failure and fracture gap displacement within the first 100 cycles. Our difference for cycles to failure was significant with a total number of 1000 cycles, and thus considered sufficient.

Our results showed that SA only withstood 53.9% of cycles to failure compared to fractures treated with TS. Furthermore, a statistically significant correlation between BMD and cycles to failure in the SA group could be shown. O’Donnell et al. [24] as well as the few previously published biomechanical studies comparing SA and TS in patella or quadriceps tendon repair [10, 11, 13, 35, 39, 40] did not evaluate the parameter of cycles to failure, perhaps because their specimen did not fail during their mostly significantly lower number of cycles. The impaired anchors’ performance in comparison to TS observed in our study could be due to their threads, which can lead to stress concentration in the bone structure, resulting in a reduced pull-out resistance capacity. Furthermore, the anchors are placed in the spongiosa, while the load-bearing parts of the TS are placed over the harder bone and corticalis of the superior pole of the patella [41]. As the BMD of the specimen in the SA group significantly correlates with the cycles to failure, an amplification of this effect can be assumed in more osteoporotic specimens. Their failure mechanism supports this assumption, as most anchors were pulled out of the bone (Fig. 3a) and different studies have already shown the direct correlation between anchor pullout and BMD [42, 43]. Hence, TS can be considered especially in elderly and osteoporotic patients. However, our data have to be interpreted with caution because of the low number of specimens. Other studies with perhaps only osteoporotic specimens must follow to corroborate our findings.

Secondly, the SA group withstood significantly lower loads to failure of 51.2% than the TS. The stress concentration at the anchor threads in the spongiosa can be considered as a reason for the inferior performance in load to failure tests. In contrast, O’Donnell et al. [24] showed a comparable maximum load to failure between SA and TS group, although their instrumentations only withstood markedly lower loads. This may be due to their setup of only patellae with patella tendons compared to our more physiological setup of whole knees. The fact that only explicitly non-osteoporotic specimens with a T-score > − 1.0 in dual-energy X-ray absorptiometry (DEXA) were included in their experiments supports our theory that SA need a sufficiently higher BMD for stable osteosynthesis in comparison to TS. In addition, their SA failures occurred mostly at the suture–anchor interface as opposed to anchor pullout in our tests. The authors attribute this mainly to their single-loaded anchors with only one suture attached instead of double-loaded ones like in our setup. In our case, the connection between osteoporotic bone and anchor seem to be the least resilient spot.

Although no significant difference in fracture gap displacement was detected in this study, a trend towards smaller fracture gap displacement with increasing number of cycles was observed in the SA group compared to the TS group. Our observations are consistent with those of O’Donnell et al. [24], as well as of Bushnell et al. [39] and Ettinger et al. [35] in patella tendon repair, who all three reported significantly smaller gap formation after 250 cycles for SA than TS. Bushnell et al. [39] explained the higher gap formation of TS with its greater “dead length” through the patella bone, resulting in increased elastic stretching. Krushinski et al. [44], referring to these studies, hypothesized the main cause for the lengthening in the suture and soft tissue interface as clinically extensive gapping occurred in both the SA and TS groups. They proposed pretensioning of the Krackow stitches, resulting in a significant reduction of fracture gapping of TS in their publication, but with no clinical relevance. Our results may also be attributed to the lack of suture of the capsule and skin compared to the clinical situation, as both can have an important influence on the extensor mechanisms’ stability, especially in the first weeks of recovery [45]. Altogether, a 1:1 correspondence to the clinical situation is not possible, but SA might be able to provide and sustain a more anatomical reapproximation and more rigid fixation of inferior pole patella fracture as for patella tendon repair [39].

## Conclusions

The biomechanical results of our study suggest that the stability of TS is still preferable to SA in osteoporotic patients, as SA showed inferior results compared to TS with respect to the number of cycles and destructive load to failure. On the other hand, if the bone provides enough stability, the possibly more rigid fixation by SA may again prove beneficial in combination with the many clinical advantages of SA. Considering the existing literature and our clinical experience, we recommend that the surgical strategy should be adapted to the individual case. BMD measurement is likely an important selection criterion for choosing the individual surgical approach in elderly patients. This also includes the possible combination of various surgical procedures. Further biomechanical and clinical investigations will be necessary to determine whether SA can be a viable or even better alternative to TS, especially in non-osteoporotic bone.

## Data Availability

All data generated or analysed during this study are included in this published article. The datasets on cycles to failure, fracture gap and load to failure as well as demographic data on the specimens have been deposited in the RWTH Publications repository, https://doi.org/10.18154/RWTH-2022-02362 [46]. Raw data of the electromagnetic tracking system are not publicly available due to their huge amount of data, but are available from the corresponding author on reasonable request. Their essential information has been summarized in the cycles to failure data. Requests for material should be made to the corresponding author.

## Abbreviations

SA: Suture anchor fixation
TS: Transosseous suture fixation
BMD: Bone mineral density
